# Treatment of Enlarged Spleen (Ague Cake)

**Published:** 1872-05

**Authors:** 


### TREATMENT OF ENLARGED SPLEEN (AGUE CAKE).

Dr. Thomas Hill (Richmond and Louisville *Medical Journal*) reports the following cases:

K. P.—Aged eight months, male. This little child had been having ague and fever almost from his birth, his mother having suffered with them almost through her whole pregnancy. The whole family having ague and fever, living on a low mill-pond, I recommended a removal to a more healthy locality, and put them on quinine and iron. They all soon improved, except the baby. Upen carefully examining it I detected the presence of a large ague cake, and made the following prescription:

| | |
|---|---|
| R.—Quinine, . . . . . . | grs.viii. |
| Hyposulph. Soda, . . . . | grs.xiv. |
| Water, . . . . . . | oz.j. |
| Elix. Vitriol, . . . . . | gtts.vi. |

Sig.—Give a teaspoonful every two hours. A poultice of hoarhound to be applied to the spleen. In two days the enlargement had subsided, and by the use of small doses of iodide of iron, the child was soon restored to health.

Case 2d was an adult male. In this case ten-grain doses of hyposulphite of soda, with one grain of quinine every four hours, completed the cure.

